# Evaluation of a laboratory capacity strengthening project: a case of the summative assessment of the African Field Epidemiology Network (AFENET) laboratory project 2010 - 2016

**DOI:** 10.11604/pamj.2018.30.297.15693

**Published:** 2018-08-30

**Authors:** Humphrey Kabugo, Davis Ashaba, Fausta Mosha, Rebecca Babirye, Christine Kihembo, Mercy Maeda, Kerine Hay, Olivia Namusisi, Peter Nsubuga

**Affiliations:** 1 African Field Epidemiology Network, Kampala, Uganda; 2 National Health Laboratory Quality Assurance and Training Center, Tanzania; 3 Global Public Health Solutions, Atlanta, GA, USA

**Keywords:** Evaluation, laboratory strengthening, evaluation design matrix, Word cloud, AFENET

## Abstract

**Introduction:**

Between September 2010 and September 2016, the African Field Epidemiology Network (AFENET) implemented laboratory strengthening initiatives through a cooperative agreement with the International Laboratory Branch of the US Centers for Disease Control and Prevention (CDC). This project aimed at improving laboratory Quality Management Systems (QMS) towards accreditation in Africa and the Caribbean region and was implemented in 11 countries in the Caribbean and seven African countries. This paper describes the results of a summative evaluation that was commissioned at the end of the project.

**Methods:**

The evaluation team comprised an external consultant who led the evaluation design and implementation and AFENET project staff. The evaluation was done in all 11 Caribbean and seven African countries where the project was implemented. We formulated three evaluation questions to focus and guide the exercise: 1) Were project activities implemented as originally intended? 2) Did the project achieve the objectives it was intended to accomplish over its life? 3) Are the impacts of project interventions likely to survive in the long run? We developed 14 sub-questions from the three evaluation questions and obtained data using a set of online questionnaires. We conducted validation visits to six participating countries; four in Africa and two in the Caribbean.

**Results:**

Out of 14 sub-questions that were used to evaluate the project, six (43%) were fully achieved, six (43%) were partially achieved, and two (14%) were not achieved. In effect, > 80% of the sub-questions were either fully achieved or partially achieved. The most frequently mentioned success was the introduction of QMS in participating laboratories, which led to quality improvement in laboratory processes, participation in SLMTA (Strengthening Laboratory Management Towards Accreditation)/SLIPTA (Stepwise Laboratory Quality Improvement Process Towards Accreditation) and attainment of accreditation by some of the project laboratories. However, there were neither clear plans nor budget lines to mainstream activities that were supported under the project into regular activities of the ministries of health of participating countries.

**Conclusion:**

The evaluation team concluded that there were adequate numbers of laboratorians trained in the FELTP laboratory track but only in Kenya. The DTS testing and biosafety programs were implemented and expanded in participating countries. HIV laboratory networks were strengthened in all participating countries and laboratory information systems were implemented in the Caribbean countries, but the basic laboratory information systems in the African countries were not implemented beyond pilot stages. There were no clear plans and budget lines provided by respective ministries of health to mainstream the activities that were supported under the project. The evaluation team recommended that AFENET develops a new laboratory strategic plan that could leverage the activities that were funded and implemented in the project.

## Introduction

Recently, there has been a growing concern about the impact of development assistance that has rekindled interest in assessing how well development projects and social programs have been meeting their objectives [1]. Evaluation is a systematic and objective assessment of an on-going or completed project, program or policy, its design, implementation and results. The aim of conducting a program evaluation is to determine the relevance and fulfilment of its objectives, efficiency, effectiveness, impact and sustainability [2]. Program evaluation drives organisational learning based on lessons learned from the work evaluated. It also serves as an input to provide decision-makers with knowledge and evidence about performance and good practices, present and future planning and strategies and policies by providing targeted recommendations to project managers [2]. Between September 2010 and September 2016, the African Field Epidemiology Network (AFENET) implemented laboratory strengthening initiatives through a cooperative agreement with the International Laboratory Branch of the US Centers for Disease Control and Prevention (CDC). This project aimed at improving laboratory Quality Management Systems (QMS) towards accreditation in Africa and the Caribbean region [3, 4] and was implemented in 11 countries in the Caribbean and seven African countries. The project specifically worked through enhanced and expanded Laboratory Quality Assurance (QA) for HIV rapid testing; biosafety training and biological safety cabinet maintenance, QMS training and mentorship and training laboratorians through Field Epidemiology and Laboratory Training Programs (FELTPs), in addition to other laboratory management strengthening activities. The project was implemented in 11 countries in the Caribbean (i.e., the Bahamas, Barbados, Dominica, Grenada, Jamaica, St. Kitts and Nevis, St. Lucia, St. Vincent and the Grenadines, Suriname and Trinidad and Tobago) and seven African countries (i.e., Angola, Ethiopia, Cameroon, Kenya, Swaziland, Tanzania, and Uganda) [5, 6]. AFENET provided technical, logistical and other support to the countries and public health laboratories, leveraging on its existing collaborations with Field Epidemiology and Laboratory Training Programs (FELTPs) in the Africa region; various ministries of health, CDC headquarters and country offices and other partners [7]. The project ended in September 2016, necessitating a summative evaluation of the activities, to document any lessons learned from the project and to guide AFENET management and the project stakeholders on next steps.

## Methods

### Evaluation setting

The evaluation took place between January and May 2017, starting three months after the end of the project. The evaluation team comprised an external consultant who led the evaluation design and implementation and AFENET project staff both in the Caribbean and in Africa. All 11 countries in the Caribbean and seven African countries where the project was implemented were targeted in the evaluation. We held weekly consultative meetings to discuss the design of the exercise and refine logistical arrangements.

### Purpose of the evaluation and evaluation questions

The purpose of the evaluation was to systematically document the effectiveness of project implementation and to identify lessons learned during implementation for use in future programming. We formulated three evaluation questions to focus and guide the evaluation: 1) Were project activities implemented as originally intended? 2) Did the project achieve the objectives it was intended to accomplish over its life? 3) Are the impacts of project interventions likely to survive in the long run?

### Literature review

We reviewed relevant literature such as the project proposal, annual reports, notice of awards and annual work plans to inform the evaluation. The evaluation was guided by the PEPFAR evaluation standards of practice [8]. Based on the reviewed literature, we developed an inception report to spell out the timelines of evaluation activities, selected key informants, countries selected for validation visits, data collection tools and the final report outline. A shared project dropbox folder was set up to allow for easy sharing of evaluation materials among the team members.

### Evaluation design

We employed a mixed quantitative and qualitative evaluation approach. We developed an Evaluation Design Matrix (EDM) to guide and focus the evaluation [**2**]. The EDM had three themes addressing the three evaluation questions as described above. For each of the themes, the Evaluation Design Matrix had the following elements: evaluation questions, sub-questions, elements of interest, indicators, targets and data sources.

### Data collection

We prepared, validated and administered three web-based tools to collect data. The first tool was for the AFENET project team to identify key informants in the countries and their contact information. The second tool was for abstraction of key project documents, while the third tool was administered to country key informants, stakeholders and project participants. The focus for the third tool was on the main successes, challenges, best practices, lessons learned and opportunities of the project. The third tool was sent out to respondents in all 11 countries in the Caribbean and seven African countries where the project was implemented. All respondents were participants in the project and their responses were returned directly to the evaluation team lead for synthesis.

### Country validation visits

We selected six countries for in-country visits and meetings with key informants and stakeholders to verify the information obtained from respondents using the online tools. These countries were selected based on the amount of funding received for project activities, availability of key informants and in-country staff previously employed by AFENET to support the evaluation effort and lastly the number of activities that were implemented in that country. A two-person evaluation team visited each of the six countries. The countries that were visited to validate the online findings were Angola, Barbados, Jamaica, Swaziland, Tanzania and Uganda.

### Data analysis

We calculated frequencies and proportions for quantitative variables using MS Excel and developed word clouds and themes for qualitative variables.

### Report writing

A draft evaluation report was prepared and shared with the AFENET project team for input and eventually presented to project stakeholders at a dissemination meeting in Kampala, Uganda. The draft report was turned into a final report for the stakeholders and project team to implement the evaluation recommendations. For each of the six countries that were visited, specific reports were prepared to provide country-specific findings and recommendations.

### Ethical consideration

This was an evaluation exercise for a laboratory systems strengthening project which had ended and thus did not require approvals from ethical review boards. The aim of the evaluation was fully explained to all respondents.

## Results

A total of 40 respondents filled out the online questionnaires. To determine whether project activities were implemented as intended initially, eight sub-questions were assessed. Of these eight, four (50%) were fully achieved and three were partially achieved (Table 1, Table 1 (suite)). The fully achieved sub-questions were on adequate numbers of personnel trained, implementation and expansion of the Dry Tube Specimen (DTS) proficiency testing program, strengthening of laboratory capacity through SLMTA and development of national laboratory strategic plans. The partially achieved sub-questions were on the biosafety training program through FELTPs and the biosafety cabinet certification program and this was reportedly due to lack of approved funding. Overall, project coordination and project monitoring and evaluation were also partially achieved.

**Table 1:** Summary evaluation results on theme one: Implementation

Evaluation Question: Were AFENET laboratory project activities implemented as originally intended?						
Sub-questions and context	Elements of interest	Indicators	Target	Data source	Achievement evaluation subquestions Yes/No/Partial	Summary of results
*Were adequate numbers of personnel trained at master’s degree level in laboratory management and policy using the available FELTP curriculum?*	-Number of personnel trained to master’s degree level -Skills gained in laboratory management and policy -Adequacy of the numbers trained	-Number of personnel with master’s degree level training in laboratory management and policy	20	Annual, quarterly FELTP and project reports	**Yes**	The project supported a laboratory resident advisor in Kenya FELTP from September 2012 to September 2015. 51 residents were enrolled in the laboratory track of the Kenya FELTP during the project period.
*Were the Dry Tube Sample (DTS) proficiency testing technique expanded and implemented in selected PEPFAR-supported countries adequately?*	-Targeted number of labs with capacity to produce DTS -Targeted number of testing sites enrolled in EQA using DTS -Extent of expansion of the DTS technique.	-Number of laboratories with capacity to produce DTS	16	Project reports	**Yes**	HIV EQA using DTS was expanded and implemented in Angola, Cameroon, the Caribbean, Swaziland, Tanzania and Uganda.
		-Number of HIV testing sites enrolled in EQA using DTS	700			
*Was the WHO Biosafety Training Program developed and implemented through FELTPs appropriately?*	-Existence of evidence of the developed program -Evidence of implementation of the training program -Evidence of capacity building	-Number of personnel competent in WHO biosafety practices	50	Project reports (annual, quarterly), Continuation application documents	**Partial**	Funds were only provided to support a standalone biosafety course in Kenya FELTP. Funding was also used to develop occupational safety and health guidelines.
		-Number of FELTPs offering training	1			
*Was a biological safety cabinets certification program developed and implemented through FELTPs appropriately?*	-Targeted number of personnel capable of certifying biological safety cabinets -Evidence of development of program -Evidence of capacity building in certification	-Number of personnel capable of certifying biosafety cabinets	45	Project reports (annual, quarterly reports) Continuation application documents	**Partial**	No funds were provided to implement biosafety cabinet certification through the FELTPs. Tanzania, Uganda, Rwanda, and Ethiopia initiated biosafety cabinet certification and laboratory equipment maintenance. Tanzania and Uganda followed through with the partial training of biosafety cabinet certification engineers. Only 7/45 engineers were trained.
		-Number of trained personnel on certification	20			
		-Number of biosafety cabinets certified	40			

Implementation: Yes =4, Partial = 4, Total sub questions 8, Mark 50%

**Table 1 (suite):** Summary evaluation results on theme one: Implementation

Was laboratory capacity (equipment procurement and service) strengthened through SLMTA?	-Trained laboratory personnel in each country -Participation in QMS by each country	Number of laboratory personnel trained.	50	Project reports (annual, quarterly reports), Continuation application documents	Yes	Several pieces of laboratory equipment were purchased for the participating laboratories. SLMTA was seen as one of the best programs that the project carried out, with potentially long-lasting effects in the participating countries. Five (50% of targeted 10) laboratories were internationally accredited under ISO 15189:2007 10 laboratories were certified with stars 1-3 under WHO SLIPTA.
		Number of labs enrolled into QMS and laboratory accreditation program	20			
		Number of labs mentored	20			
		Number of labs accredited.	10			
		Number of laboratories with 1-3 star rankings	10			
Were national laboratory strategic plans (NLSP) developed in all project countries?	Existence of Strategic plans	Number of NLSPs developed	10	Project reports, Continuation application documents	Yes	11 NLSPs were developed in the Caribbean (the Bahamas, Barbados, Dominica, Grenada, Jamaica, St. Kitts and Nevis, St. Lucia, St. Vincent and the Grenadines, Suriname, and Trinidad & Tobago).
Were the various interventions coordinated appropriately?	Monthly or quarterly team meetings	Number of team meetings with minutes	At least 4 per year	Reports	Partial	There was central coordination from the AFENET Secretariat and regular reporting to CDC. However, over the life of the project, the implementers never had a project meeting, and the key implementers and collaborators never reached the Secretariat.
Was there a monitoring and evaluation (M&E) plan?	-M&E plan available. -Was the plan used?	-Written M&E plan & database -Written decisions taken based on M&E results		Written plan	Partial	There was no written M&E plan, but annual reports were prepared according to the objectives of the project which implied some form of tracking of outputs.

Implementation: Yes =4, Partial = 4, Total sub questions 8, Mark 50%

Three sub-questions were assessed to determine whether the project achieved its objectives. Of these, two (67%) were fully achieved and one was partially achieved (Table 2). The objectives that were fully achieved were strengthening the HIV network and laboratory quality management systems in participating countries. Implementation and expansion of an easy to use laboratory information system (LIS) in Kenya, Uganda, Tanzania and the Caribbean was partially achieved. The Basic Laboratory Information System (BLIS) did not work in the countries where it was introduced, while the Caribbean region used a commercial laboratory information system.

**Table 2:** Summary evaluation results on theme two: effectiveness

Evaluation Question: Did the AFENET laboratory project achieve its objectives?						
Sub-questions and context	Elements of Interest	Indicators	Target	Data Source	Achievement of evaluation sub-questions Yes/No/Partial	Summary of Results
*Was the HIV network (viral load, drug resistance, EID, PIMA^TM^) strengthened in the selected Countries*		-Number of countries supported to develop HIV network frameworks	2	HIV network frameworks Continuation application documents Project reports	**Yes**	The HIV networks were created and strengthened within the participating countries
		-Number of participating sites	20			
*Was an easy to use Laboratory Information System (LIS) expanded and implemented in Kenya, Tanzania, Uganda and the Caribbean?*	-Targeted number of laboratories installed with easy-to-use LIS -Capacity building in managing the LIS -Number of countries that benefitted	-Number of laboratories installed with easy-to-use LIS	23	Project reports (Annual, quarterly reports) Continuation application documents	**Partial**	The LIS was only developed in the Caribbean using proprietary tools. BLIS did not seem to work in the countries where it was expected to work. Only 10/23 laboratories eventually had LIS.
		-Number of LIS equipment procured	10			
		-Number of IT persons trained	20			
*Were Laboratory Quality Management Systems strengthened in the participating countries*	-Strengthened QMS systems In all project countries	-Number of QMS related training conducted	8	Project reports (Annual, quarterly reports) Continuation application documents	**Yes**	QMS systems were introduced and strengthened in all the participating countries
		-Laboratory related documents the laboratories were supported to develop	15 documents per lab			
		-Equipment service contracts in place	3			

Effectiveness: Yes = 2, Partial = 1, Total sub questions = 3, Mark =67%

To establish whether the results of the project interventions were likely to be sustainable, three sub-questions were assessed. Of these, one was partially achieved and the other two were not achieved (Table 3). The partially achieved sub-question was on the presence of clear plans for mainstreaming the results of the project in the participating countries. Whereas no plans were present, stakeholders in the countries indicated that they would mainstream the results when the project funding ended. However, there were no plans to provide ongoing mentoring of the trained staff after project funding and no budgets for mainstreaming in any of the countries.

**Table 3:** Summary evaluation results on theme three: Sustainability and Mainstreaming

Evaluation Question: Are the impacts of project interventions likely to survive into the long run?						
Sub-questions and context	Elements of Interest	Indicators	Target/Deliverable	Data Source	Achievement evaluation sub-questions Yes/No/Partial	Summary of Results
*Was there a plan to mainstream the project activities into regular MOH services*	Identification of potential mentors Incorporation into pre-service training Stakeholders’ views on mainstreaming	A written plan for mainstreaming	Plan	Stakeholders	**Partial**	No plans were evident, but some countries indicated that they would try to mainstream the activities after funding ended.
*Was there a plan to provide ongoing mentoring for the trained participants?*	Training of region-based facilitators	A written plan for ongoing mentoring	Plans	Stakeholders	**No**	
*Was there a budget for mainstreaming?*	Budget line for QMS and EQA activities for the participating countries	Approved budget line	Approved budget lines		**No**	

Sustainability and Mainstreaming: Yes =0, Partial =1, No =2, Total sub questions = 3 Mark 0%

In summary, the three evaluation questions were segmented into 14 sub-questions that were used to focus the evaluation of the project. Out of the 14 sub-questions, six (43) were fully achieved, six (43%), were partially achieved and two (14%) were not achieved. The most frequently mentioned success of the project was the introduction of quality management systems in participating laboratories which led to quality improvement in laboratory processes. Other successes were the participation in SLMTA (Strengthening Laboratory Management Towards Accreditation)/SLIPTA (Stepwise Laboratory Quality Improvement Process Towards Accreditation), attainment of accreditation by some laboratories, establishment of an external quality assurance scheme for HIV rapid test kits using Dry Tube Specimen (DTS) and participation of laboratories in outbreak response.

**Figure 1:**
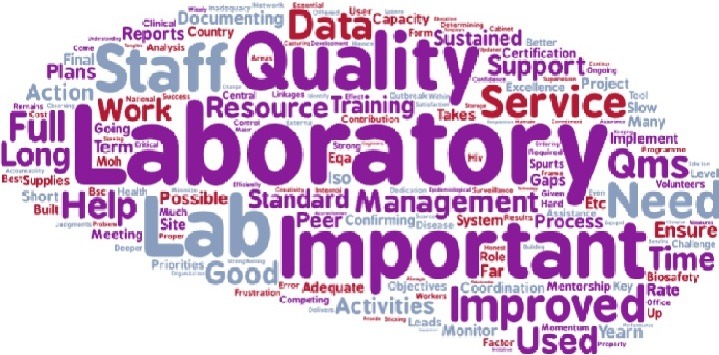
Word cloud of the lessons learned in the implementation of the AFENET laboratory project

**Figure 2:**
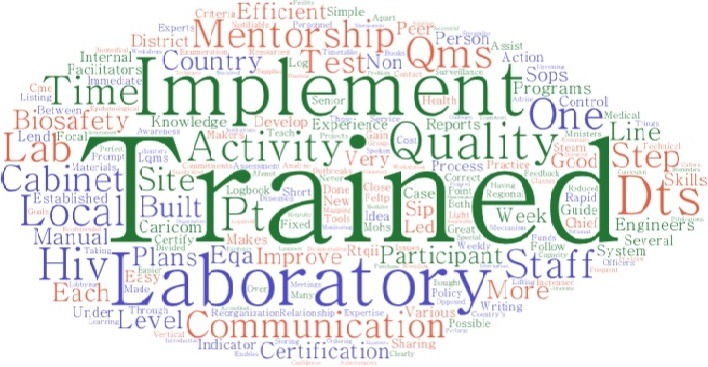
Word cloud of the best practices from the AFENET laboratory project

**Figure 3:**
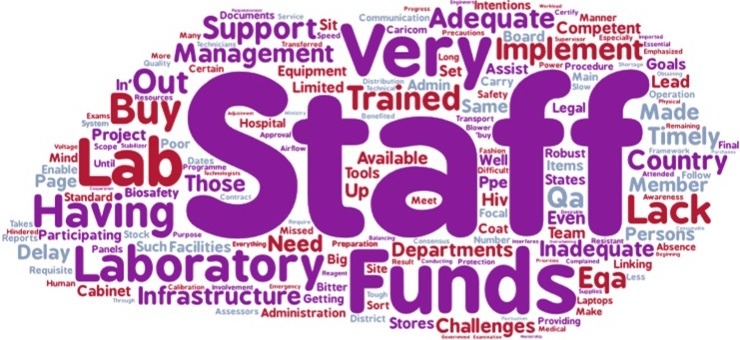
Word cloud of challenges faced during implementation of the AFENET laboratory project

One of the main lessons learned from project implementation was the importance of laboratory quality management systems (Figure 1). Among the top five best practices from the project that were identified were effective mentorship, training of laboratory staff, biosafety, QMS and DTS systems (Figure 2). Challenges that were identified include shortage of staff (worsened by transfers of staff that had been trained by the project), lack of “buy-in” by some stakeholders in the laboratory change process, for instance laboratory managers and lack of funding for infrastructural changes that were beyond the scope of the project (Figure 3).

## Discussion

In this evaluation of the 6-year project which was implemented in several countries in Africa and the Caribbean, we found that out of the 14 sub-questions, six (43%) were fully achieved, six (43%) were partially achieved and two (14%) were not achieved. In effect, > 80% of the sub-questions were either fully achieved or partially achieved. During the implementation of the project, adequate numbers of laboratorians were trained at master’s level through Field Epidemiology and Laboratory Training Programs (FELTPs), particularly in Kenya. This success was possible because AFENET supports FELTPs in Africa as part of its core mission; this success also points to the viability of joint training of epidemiologists and laboratory scientist to solve public health problems in synergy [9-11].

The DTS EQA program was also expanded and implemented in selected countries adequately; this success points to the long-awaited need for cheaper, more robust methods of conducting EQA for HIV rapid test kits in developing countries [6, 12]. National laboratory strategic plans were developed for participating countries and basic laboratory equipment was supplied to participating laboratories. This shows how the project was able to support critical needs for countries, as national laboratory strategic plans are the basis for larger public health laboratory strengthening efforts.

The partial achievement of the biosafety training program through the FELTPs is of concern as this training is essential. Of more concern is the lack of funding to complete the biological safety cabinet certification program; as many countries have biological safety cabinets that are used daily and it is necessary to ensure that they are certified. The approach of biosafety training coupled with biological safety cabinet certification could have provided a local certification workforce in the participating countries.

The project strengthened HIV laboratory networks in several countries. This, along with implementation and expansion of laboratory quality management systems, led to accreditation of several public health laboratories and may be one of the long-lasting effects of the project. However, lack of plans by respective governments to provide mentorship of staff that were trained to implement the quality laboratory systems and other activities that were supported, beyond the project life is a major concern. This finding coupled with lack of clear budget lines for mainstreaming from the participating countries may dilute the effects of this project in the medium to long-term.

A best practice is something that was done well and can be shared with others, it is easy to do and does not require a lot of resources but leads to a sustainable impact [13]. The top five best practices from the project that were identified were effective mentorship, training of laboratory staff, biosafety, QMS and DTS systems. The successes as described by the stakeholders were in staff capacity development, implementation of laboratory quality management systems, development of the HIV EQA systems and accreditation of several laboratories. These successes laid the foundation for public health improvements in the countries that were part of the project and go beyond the HIV disease-specific funding that was used for the project. These horizontal public health capacity efforts enable categorical disease-specific funds to be used for more than the specific disease in this case HIV [14]. As expected, infrastructure, staffing and funds are the main challenges that were described by the stakeholders; perhaps a future project of this nature should involve a specific component of mainstreaming and long-term planning.

Interpretation of the results of this evaluation is subject to at least four limitations to generalisation. Firstly, all respondents were participants in the project and could have views that were influenced by their role in the project. Secondly, there may also have been a natural bias to focus on program successes although the evaluation team tried to tease out other critical points to the questions. Thirdly, although several attempts were made to obtain answers from national-level stakeholders, only a few responded, but if more had responded, their answers could have been different from those of the few who answered. Fourthly, some key informants that were contacted were not available to be interviewed due to scheduling difficulties. Finally, evaluation questions required the respondents to have adequate recall of events that occurred in the past and this could have had a bearing on the results. The evaluation team tried to triangulate sources of information to limit the effect of the various limitations and we believe that the findings provide an adequate view of what transpired in the project.

A project of this magnitude and complexity could have benefited from better coordination and an explicitly written monitoring and evaluation plan. Indeed, we found it noteworthy that some key project staff had never visited the project secretariat. Several annual reports provided evidence of project monitoring, but more could have been done.

## Conclusion

The evaluation led to the following conclusions based on the evaluation questions.

### Were project activities implemented as originally intended?

There were adequate numbers trained in the FELTP laboratory track but only in Kenya, out of a possible 12 FELTPs. The DTS proficiency testing program was implemented and adequately expanded in participating countries. The biosafety program was developed and implemented in the participating countries but not directly through the FELTPs except in Kenya. The biosafety cabinet certification program for engineers was only partially implemented, due to funding limitations. Laboratory capacity was strengthened in all participating countries through SLMTA/SLIPTA and equipment purchases. National Laboratory Strategic Plans were developed in some but not all the participating countries in the project. The various activities in the project were coordinated appropriately by AFENET secretariat and the project leadership, although the implementers of the project needed to have an annual or a biennial project meeting. There was no explicitly written project monitoring and evaluation plan although activities and outputs were tracked.

### Did the project achieve its objectives?

HIV laboratory networks were strengthened in all participating countries. Laboratory information systems were implemented in the Caribbean countries, but the basic laboratory information systems in the African countries were not implemented beyond pilot stages. Quality management systems were strengthened in all the participating countries in the project.

### Are the impacts of project interventions likely to survive in the long run?

There were no clear plans and budget lines provided by respective ministries of health to mainstream the activities that were supported under the project. There were also no plans to provide ongoing mentorship of their staff that had been trained by the project. However, other CDC implementing partners in some of the countries may undertake some of these tasks.

### Recommendations

The evaluation team made the following recommendations: 1) AFENET should develop a new strategic plan to guide the organisation’s laboratory activities. In this plan, AFENET should proactively look for opportunities to continue the work that was funded under this project because activities may end without additional funding. For example, partnering with the country and regional stakeholders to develop new projects that could be submitted to in-country and international funding partners. 2) AFENET should explore mechanisms to continue a mentorship relationship with individuals who were trained during the project. For example, setting up distance learning methods or electronic communication. 3) AFENET should try to evaluate opportunities for linking SLMTA training to the existing FELTP training. FELTP laboratory track participants can provide a pool of assessors and mentors for their respective countries and regions.

## Competing interests

The authors declare no competing interest.
